# Treatment of Pulmonary Tuberculosis by Artificial Pneumothorax, Showing Author’s Apparatus

**Published:** 1911-06

**Authors:** S. T. Harris

**Affiliations:** Valdosta, Ga.


### TREATMENT OF PULMONARY TUBERCULOSIS BY ARTIFICIAL PNEUMOTHORAX, SHOWING AUTHOR'S APPARATUS.

By Dr. S. T. Harris, Valdosta, Ga.

The status of artificial or induced therapeutic pneumothorax will in part be shown by this study, which is based on the findings of various authors, citing in their reports over 300 cases.

In proof that the theory of artificial pneumothorax in the treatment of phthisis has a basis of scientific fact, I present the following:

### Animal Experimentation.

Reubel, to show the action of functional rest of the lung on the extent and course of pulmonary tuberculosis, partially immobilized the lungs of rabbits and dogs by wiring the ribs in such a manner as to obliterate the interspaces, thus hampering the excursions and causing this side of the chest to lag behind in respiration. He injected tubercle bacilli into a vein in the ears of these animals. Uninfected animals and a dog infected by injecting T. B. into the lung tissue were used as controls. All of his experiments confirm the fact that functional rest of the lung favored transformation of the tubercle into cicatricial tissue and caused a pronounced retrogression of the infiltration of tuberculous inflammation. The blood supply to the lung and the dissemination of bacteria from it seemed to be in direct proportions to the respiratory excursions and the activity of the movements of the lung.

Dunin, in the course of animal experimentation, has also shown the tendency of compression to produce fibrosis.

### Effects of Pathological Pneumothorax on Tuberculosis of the Lung.

These experiments on animals confirm the observations of the diseased human lung, in which the results of functional rest obtained by pathological pneumothorax and effusions have been noted for two or three centuries. That these results are curative and arresting in their action on pulmonary tuberculosis is a matter of medical history.

As an example of this fact, I beg to call your attention to the following case reported by Thue:

A young man with tuberculosis of the left lung had been under treatment for some months when hydropneumothorax developed in the diseased side and the patient began to improve. For twelve years afterwards he was in comparatively good health and doing hard manual labor. He died from a sudden hemorrhage. The autopsy showed the left lung still compressed by the pneumothorax and the tuberculous process had become obliterated. Now, in this case, we would by no means expect such results as would be obtained from the systematic application of compression. Still the course and autopsy showed that there was a clinical recovery and an anatomical cure.

### Curative Effects of Induced Pneumothorax on Conditions Other Than Phthisis.

To show that induced pneumothorax is in itself a curative measure, it would not be amiss to cite cases where favorable results have been obtained from its use in other conditions than phthisis.

Molon has been using oxygen in the pleural cavity to replace the effusions from pleurisy, with favorable results. He has treated six cases of secondary pleurisy without effusions where all symptoms disappeared after the injection of about 400 c. c. of oxygen. The symptoms returned in one case but yielded to the second injection.

In two out of three cases of Wenckebach remarkable benefit was displayed from the evacuation of tuberculous empyema and introducing air into the closed chest. Patients were in a threatening condition when the pus was aspirated in amounts up to two liters. The injections of air were repeated in from three to six weeks. At the end of 11 weeks in the first case there was neither trace of pyrothorax or pneumothorax; as also after 15 weeks in the second case. The third case was less favorable.

Forlanini reports similar results from like procedure.

The above reports might be regarded as an index to a change in the technic for the evacuation of pleural effusions. In this connection the experience of Holmgren is interesting. In 17 cases he pumped air into the pleural cavity to take the place of pathological effusions. The effusions, even to the last drop, can thus be forced out by the pressure of the instreaming air. He regards this technic as having the double advantage of evacuating the pleura and inducing a therapeutic pneumothorax when indicated.

Da Gradi reports three cases from Foralnini's clinic at Pavia where associated laryngeal lesions healed completely under pneumothorax. In his opinion, we should not regard laryngeal tuberculosis as a local process, but that it is maintained from lesions lower down by the passage of sputum.

Courmant indicates that benefit is obtained in the larynx when complicated from immobilization of the lung. While these effects in the cure of laryngeal tuberculosis are secondary, they show the remarkable effects of pneumothorax on the lung below.

A case of croupous pneumonia followed by abscess in the lung of 6 years standing is reported by Foralnini. This case had been rebellious to all methods, and was cured in a few months by artificial pneumothorax. The cure had persisted for three years up to the time of the report.

### Does Artificial Pneumothorax Cure Pulmonary Tuberculosis?

If we are to accept as true the cases and findings of those who have engaged in this work no one could fail to agree that artificial pneumothorax *can* and *does cure* pulmonary tuberculosis. No unfavorable authoritative comment has presented itself.

In reading detailed reports one is struck with the consistent uniformity of the course of cases after operation. Thus we are shown that we are dealing with a method which produces *specific, curative* results. The action of no drug nor the effects of any surgical operation present a more classical picture.

Thue gives in detail 11 cases of induced pneumothorax and the results of 13 cases of Saugman. Such advanced cases were treated that cure could not be expected although the results were encouraging. The results obtained by Bauer and Kraus in 6 cases showed that therapeutic pneumothorax may prove successful in a certain class of severe cases when otherwise there would be no recourse.

Von Muralt cites 10 cases, 7 of them regarded as hopeless, where, as a last resort induced pneumothorax was applied. He stated that one was completely cured and all the others on the road to recovery.

Spengler, out of 40 cases had favorable results from 25, transient benefit in 6, slight benefit in 6, and bad in three. He recently reports that 15 of his cases can be regarded as clinically cured. After the lapse of nine months to four years after discontinuing the treatment the patients are in splendid working condition, with no fever, cough or sputum.

Of the 102 cases which have been operated on by Brauer and Spengler, a number were cured where, in their opinion, the condition was such as to preclude cure from other methods.

Holmboe reports his results in the treatment of five cases of moderately advanced pulmonary tuberculosis where induced pneumothorax gave wonderful improvement after sero-therapy had failed. Cough and sputum ceased and temperature became normal. In one case reported in detail the weight increased 20 pounds, and the patient was able to work several hours each day. Similar results were obtained in the other four cases.

In this connection the following case might prove of interest:

Mr. F., a young lawyer, in the summer of 1906 had a severe cold, resulting in laryngitis, had a few night sweats and became very thin, had considerable cough and expectoration, especially in the morning. During October, November and December, 1906, he led an outdoor life with no work. Got back his usual strength and weight, with cough and sputum largely reduced. Laryngitis continued till the spring of 1909, when cough and epxectoration increased and patient was much worried by working or exertion. In August, 1909, sputum was examined showing T. B. At this time he went to the mountains and became a patient in one of the best equipped sanitoriums in the country. Here, in an ideal climate with the very best of dietetic and hygienic treatment, under the care of one of the most competent physicians engaged in this work, he remained a year. A consistent application of sero-therapy had been made. His condition would improve and T. B. disappear from sputum several times, but on the slightest exertion would reappear, and patient begin to lose. In August, 1910 I did the initial operation for induced pneumothorax. The injections have been continued to the present time. In about two months after operation the patient's sputum was negative and he was back at home. And in three months from the beginning of the treatment was regularly at work and has remained so with no further signs of trouble. He writes that he considers himself a well man. He has a slight amount of expectoration and nasal catarrh, but he states that he has had as much for 10 years past.

This case appears to give a very fair index to the value of artificial pneumothorax as compared to the application of a combination of the very best of other known methods.

Lapham reports a case of very severe monolateral tuberculosis involving the whole left lung: "After 18 months in bed with failure to recover, the patient is now cooking her own meals and doing light housework after only two months of artificial pneumothorax."

The well known favorable results obtained by Murphey and Lemke in a large number of cases where massive injections were used and not kept up for any length of time, add their testimony to the efficacy of artificial pneumothorax. The still more favorable results obtained by Orlandi and Fontana where the method of Forlanini was used and the very large experience of Forlanini himself for a great number of years, all lead one to the conclusion as stated by him: *"That artificial pneumothorax can cure pulmonary tuberculosis."*

When pneumothorax is properly applied we have then beyond doubt a subjective, clinical and anatomical recovery. Autopsies have shown that every anatomico-pathological condition characteristic of tuberculosis of the lung is obliterated by induced pneumothorax. The complete disappearance of all tubercle bacilli is a common result very early in the treatment.

### Prophylactic Value.

At this point I wish to emphasize the practically perfect prophylactic value of this method of dealing with tuberculosis as a general problem. Its value is self evident, and from a public health standpoint is unequalled by any other method or combination of methods in dealing with consumptives. You are enabled to not only cure the patient, but to render him innocuous.

### To What Class of Tuberculous Patient. Is Pneumothorax Applicable?

Of course, we cannot apply induced pneumothorax to every case of tuberculosis of the lungs, as for instance, very advanced lesions in both lungs. The conditions which permit of its application are defined by Lemke as follows: "Whereas bilateral are not as favorable as unilateral cases, no matter what treatment is instituted, yet compression is not contra-indicated of the lung more advanced in the disease."

Baer and Kraus believe that success may be obtained in a certain class of severe cases when otherwise disarmed.

Both Spengler and Muralt believe it applicable only to severe unilateral cases. Thue thinks that the method should be used even in afebrile cases where the tuberculous process progresses, rebellious to other measures.

Holmgren says that it is possible to apply induced pneumothorax in cases where adhesions between the sheets of the pleura apparently contra-indicate it, by injecting physiologic salt solution under a pressure sufficient to detach the adhesions and spread apart the walls of the pleura. Then inject gas.

Accordng to Lapham the contra-indications are "Any complication sufficient in itself to inhibit recovery, such as diabetes and too great involvement of the second lung. Lesions of the upper third of the second lung are no contra-indication. Compression is indicated in any case that fails to do well after a fair trial of other methods of treatment."

The statement of Forlanini that "the precise indications are monolateral lesions of considerable extent with non-adherent pleura, no matter whether there is a lesion in the other lung or not, provided it is in the initial stage," is enlarged upon by him. He now thinks that we are justified in using pneumothorax on cases with incipient lesions. His latest triumph is in the cure of two cases of bilateral pulmonary tuberculosis in which after treating one lung under induced pneumothorax—it resuming its function—the same procedure was applied to the other lung with equal success.

The range of action of this method in the treatment of phthisis is constanty widening. Of course, there is a definite limit for its successful application. But it is likely that this has not as yet been accurately determined.

### Advances In Diagnosis.

As the diagnosis of pneumothorax is essential to its intelligent application, t is not out of place to mention the following advances:

A sign mentioned by Spadaro is a protrusion in the lower part of chest when recumbent and a depression in its place when erect.

Ayers states that X-ray examination has frequently made difficult cases clear, rendered diagnosis more exact, qualified prognosis and explained the effect of treatment."

While I gather that these conclusions were arrived at from the application of X-ray in cases of pathological and accidental pneumothorax, they well express the importance of X-ray and radiography in the diagnosis of induced pneumothorax and in keeping up with the treatment.

### THE OPERATION.

*Selection of Site for Injection.*—A point away from the cardiac area and diaphragm and, if possible, a wide intercostal space thinly covered by muscle (Lapham), and subcutaneous tissue is chosen for the injection. This point should give by auscultation and percussion indications of as near normal condition of lung and pleura as can be found.

*Preparation of Field for Operation.*—The selected point and its surroundings should be prepared in the same manner as for surgical operations, then dried with a sterile cloth. It might be well at this time to mark the exact point with tincture of iodine. A dry sterile dressing should then be applied. This should be done some little time before the operation. When ready the point for injection should be thoroughly painted with tincture of iodine.

*The Operation Itself.*—As a preliminary the patient should have been given a hypodermic of morphia for the purpose of avoiding pleural reflex (Lapham). The apparatus should have been prepared, everything being sterilized, the nitrogen jar filled and the reservoir full of fairly hot sterile water. The nitogen jar should be kept warm. The whole apparatus should have been tested to see that connections were right and the flow of gas free. The point for injection is anaesthetized with ethyl chloride by an assistant.

Now, under every aseptic precaution, the patient with arm on side to be operated on raised above head, a small incision through the skin and subcutaneous tissue into the muscle below, sufficient to admit a medium sized aspirating needle is made at the lower part of the intercostal space. The needle with its point hugging the top of the lower rib in order to avoid the intercostal blood vessels and nerves, is carefully introduced. At the same time, I suggest that the patient should be instructed to draw a deep breath, which would further widen the intercostal space, and also tend to the quicker and better formation of an inter-pleural cavity. On passing through the parietal wall of the pleura, the operator will feel a loss of resistance and immediately stop. If successful, the fact will be indicated by an inrush of air causing a whistling sound. When a pleural cavity indicator is used a successful insertion of the needle would be noted by the action of the indicator. If no sound is heard or no effect is produced on the indicator, the patient is instructed to take two or three breaths to cough. If there is still failure the needle should be moved slightly back and forth, and if this fails another point should be selected for the insertion of needle. After insertion of needle you should wait several moments to note the effect upon the patient, then the nitrogen apparatus is connected with the needle, the assistant is instructed to raise the reservoir and the injection carefully begun. Inject about 250 to 400 c. c. of nitrogen. If the patient feels a sense of oppression or discomfort stop, withdraw the needle quickly and seal the incision with collodion. Apply a tight sterile dressing.

I would like to stress the importance of this initial work, as the safety of the patient and the success of the pneumothorax very largely depends on it. Forlanini says that great care must be exercised in preventing infection of the pleura. If pleurisy occurs adhesions may develop which interfere with the pneumothorax. Secondary injections should be made with the same strict aseptic precautions in the immediate neighborhood of the initial injection. A hypodermoclysis needle is used and no incision is made. These injections are comparatively simple and largely free from danger, still every care should be observed in doing them. They should be repeated at sufficient intervals to keep up a continual progression toward perfect pneumothorax, which once obtained should be maintained.

### Amount of Gas Used.

The amount of gas injected would depend entirely on the condition of the patient, as to whether there were adhesions, infiltration of the lung and distress of the patient, or symptoms pointing to the discontinuance of the injection.

Of course the size of the thorax varies in different people as well as the pleural cavity capacity. It has been found by different ones doing the work that under the Forlanini plan of frequent, repeated injections that amounts varying from 200 to 400 c. c. would ordinarily be used. It is a question for the operator to decide. Lemke reports having used 186 cu. in. at one injecton. As the capacity of the right lung compared to the left is about in the proportion of 11 to 10, you would likely use a somewhat larger quantity in the right pleural cavity.

The ultimate object of induced pneumothorax in the treatment of phthisis must be kept in mind in considering the amounts of gas to be used and the amount of pressure to be attained and kept up. The desired condition, then, of the diseased lung is immobilization for rest; sufficient pressure to drain out the pathological contents and coat the walls of the cavities and ulcerated surfaces in order that they may heal. Also sufficient for the mechanical obstruction of the various avenues by which this infection is transmitted to other parts of the lung. It is supposed that sufficient compression to cause hyperaemia has its effect in a curative way, and further that a point is reached where the absorption of toxins is prevented. It is thought that the avenues for the escape of gas are closed by a certain degree of pressure, which if continued sufficiently may also permanently relax opposing influences. Just what this amount of compression would be might vary with the individual case. Lapham mentions a valuable sign indicative of sufficient pressure which is "a sound as of water dropping." I suggest that for the best results a sufficient degree of compression to at least be negative on deep inspiration would be required. The use of pressure gauges are certainly invaluable in the treatment. Over distention leads to various unpleasant effects and is to be avoided. Too high an intra-pleural pressure would lead to more or less displacement of various organs, as the heart, and will invade the mediastinum, causing deep-seated emphysemas and painful conditions. Perrin states that accidental subcutaneous emphysema can be a compensating process in pneumothorax.

LENGTH OF TIME INJECTIONS SHOULD BE CONTINUED.

It is likely that the injections should be continued for about a year but, of course, no fixed time could be stated for all cases. It is known that pressure has been exerted for a number of years without harmful results and also the uninjured alvoli of the lung may resume their function unimpaired after having been subjected to prolonged pneumothorax.

In cases where the lung fails to expand it seems according to both Forlanini and Wenckebach that the pleura of the spared portions of the lung fail to come in contact with the thoracic pleura. In these cases I suggest a suction process using the same injecting apparatus for pneumothorax reversed. Of course, forced respiratory efforts on the part of the patient might aid in bringing about the desired condition. Forlanini states that in certain cases "the scars of the old lesions, the overstretching of the parenchyma, the bronchial deformity, etc., may render the function of the lung difficult." He further asserts that the stretching again of the healed lung and the restoration of its funcions may make possible the necessity of a second operation."

### Fatalities.

Brauer and Spengler review five fatalities showing the blunders in technic which permitted them. They assert that air embolism is the chief danger and think that this condition from injury has been reported as reflex-spasm or eclampsia of the pleura.

### Apparatus.

I beg to present this original apparatus for use in pneumothorax work.

As nitrogen is the selected agent its production in a comparatively pure and sterile state has been a matter of no little inconvenience and waste of time. With this apparatus it is possible to produce, ready for use in a very few minutes, any desired amount of sterile nitrogen. The process is simply burning out of air its oxygen with a grain alcohol flame. As the end products of the burning or complete oxidation of ethyl alcohol are water and carbon dioxide, we would then have left after burning in a closed vessel nitrogen, water and carbon dioxide. This gaseous mixture is then drawn out of the vessel through a soluton and over parts of potassium hydroxide which absorb the carbon dioxide as well as moisture, thus leaving nitrogen. In the process of drawing off the gas it passes through a small spiral tube in a flame completely sterilizing it.

The apparatus is so arranged that you may obtain your gas either from a bell-jar or from a bottle.

This nitrogen producer by proper connections may also be used as an injecting apparatus. At the same time the sterilization of the gas and absorption of carbon dioxide is going on the resultant nitrogen may be injected. The nitrogen may be drawn off into a separate injecting apparatus and used from it if desired.

### Conclusions.

Induced Pneumothorax when properly applied:

1st. Produces specific curative results.

2nd. Is curative of certain diseased conditions of the pleura and lung.

3rd. Can and does cure pulmonary tuberculosis.

4th. Has cured when all other methods failed.

5th. Has great prophylactic value by rendering the patient innocuous.

6th. While not applicable to all cases of phthisis, has a very large range of action.

7th. Must be done under strict asepsis with correct technic.

### Bibliography.

Lemke, A. F.—Pulmonary Tuberculosis. J. A. M. A. Oct. 14, 21, 28, 1899.

Lapham, Mary E.—Artificial Pneumothorax in the Treatment of Pulmonary Tuberculosis. Journal-Record of Medicine. Atlanta, Jan. 1911.

Brauer, L. and Spengler, L.—Induced Pneumothorax in Pulmonary Tubercolosis. Beitrage zur Klinik der Tuberkulose, Wurzburg, April 1911.

Brauer and Sprengler.—Induced Pneumothorax in Pulmonary Tuberculosis. Beitrage zur Klinik der Tuberculose, Wurzburg. March, 1911.

Brauer and Spengler.—Erfahrugen and Ueberlegungen zur Lungenkollapstherapie. Beitrage zur Klinik der Tuberkulose, Wurzburg, Dec. 1909.

Spengler, L.—Induced Pneumtohorax in Severe Unilaterial Pulmonary Tuberculosis. Munchener medizinische Wochenscrift. Feb. 1911.

Spengler, L.—Therapeutic Pneumothorax. Correspondenz—Blatt fur Schweizer Aerzte. Basle. Dec., 1910.

Spengler.—Course of Pulmonary Tuberculosis under Therapeutic Pneumothorax. Correspondenz—Blatt fur Schweizer Aerzte. Basle. Dec., 1909.

Holmgren, J.—Ausblasung anstatt Aspiration von Pleuraergussen. Mitteeilunger ans den Grenzgebeiten der Med. und Chir. Jena. XXII. No. 2.

Helmgren, J.—Zur Technik der Kompressionbehandlung bie Lungentuberkulose. Munchener medizinisbe Wochenscrift. Sept. 6, 1911.

Bauer and Krans, H.—Treatment of Pulmonary Tuberculosis with Artificial Induced Pneumothorax. Weiner Klinische Wochenschrift. Vienna. April, 1910.

Forlanini, C.—Artificial Pneumothorax. Deutsche medizinische Wochenschrift. Jan., 1911.

Forlanini, C.—Fall on seit 6 Jahren bestenden durch kunstilchen Pneumothorax behandeltem Lungenabszess. Munchener medizinische Wochenschrift. Jan., 1911.

Forlanini, C.—Artificially Indcued Pneumothorax on Both Sides. Deusche Medizinische Wochenschrift, Berlin. Jan., 1911.

Spengler, L.—Permanent Cure of Tuberculosis with Induced Pneumothorax. Munchener medizinische Wochenschift. Feb., 1911.

Thue, K.—Treatment of Pulmonary Tuberculosis with Artificial Pneumothorax. Worsk Magazin for Laegevidenskaben Christiana. Feb., 1909.

Von Muralt, L.—Treatment of Severe Unilateral Pulmonary Tuberculosis with Surgical Pneumothorax. Menchener medizinische Wochenschrift. Jan., 1910.

Molon, C.—Treatment of Pleurisy by Artificially Induced Oxygen Pneumothorax. Gazetta degli Oppedali e delle Cliniche. Leipsic. March, 1910.

Wenchebak, K. F.—Healing of Tuberculous Process in Lung under herapeutic Pneumothorax. Mitteilungen ans den Granzebeiten der Med. und. Chir. Jena. April, 1909.

Courmant, J.—Treatment of Pulmonary Tuberculosis by Artificially Induced Pneumothorax. Medical. Lyons. Dec., 1910.

Ayers, J. B.—Analysis of Seventy-two Cases of Pneumothorax. Boston Med. and Surg. Journal. Sept. 29, 1910.

Da Gradi, A.—Verlauf der Kehlkopftuberkulose bei der mit kunstlichen Pneumothorax behandelten Lungenscwindsucht Deutsche medizinische Wochenschrift Berlin. June, 1910.

Reubel, C. A. N.—Action of Functional Rest of the Lung on the Extent and Course of Pulmonary Tuberculosis. Beitrage zur Klinik der Tuberkulose, Wurzburg, 1910.

Holmboe, W.—Artificial Pneumothorax in the Treatment of Pulmonary Tuberculosis. Lancet, London. Sept., 1910. Aug, 1910.

Perrin, M.—Accidental Subcutaneous emphysema in two Cases of Valve Pneumothorax, a compensating process. Archives generales de Medicine. Paris. April, 1910.

Spadaro.—Un nuovo segno fisico dello pneumotorace. Policinico, Rome. Oct. 3, No. 118.

